# Dual-mode multichannel imaging system for high-throughput live-cell monitoring across large fields of view

**DOI:** 10.1117/1.JBO.30.12.126006

**Published:** 2025-12-12

**Authors:** Christos Katopodis, Dimitris G. Papazoglou, Ioanna Zergioti

**Affiliations:** a National Technical University of Athens, School of Applied Mathematical and Physical Sciences, Athens, Greece; b University of Crete, Department of Material Science and Engineering, Heraklion, Greece; c Foundation for Research and Technology-Hellas (FORTH), Institute of Electronic Structure and Laser, Heraklion, Greece

**Keywords:** optics, multichannel imaging, organ-on-a-chip, high-throughput microscopy

## Abstract

**Significance:**

High-throughput live-cell imaging is crucial for biological applications, including organ-on-a-chip (OoaC) platforms, yet conventional optical systems face a fundamental trade-off between magnification and field of view (FOV). This limitation hinders the ability to capture large-scale biological dynamics while maintaining single-cell resolution. We address this gap by introducing a scalable, high-resolution imaging solution specifically tailored for OoaC platforms and other microfluidic-based systems.

**Aim:**

We aim to develop a dual-mode multichannel optical imaging system capable of achieving single-cell resolution over an extended FOV while maintaining a working distance suitable for integration with microfluidic devices.

**Approach:**

The system employs microlens arrays in conjunction with laser-fabricated micro-aperture arrays to optically isolate imaging channels, minimizing crosstalk. Two operational modes are implemented: (1) rapid sampling mode for instantaneous, partial-area imaging and (2) full-field imaging mode, utilizing micro-scanning and computational stitching to generate a seamless high-resolution composite. The system’s performance was validated through experimental imaging and theoretical modeling.

**Results:**

The system achieves an FOV of $8.4\times 6\;mm^{2}$ at 4× magnification with single-cell resolution while preserving a 14 mm working distance. Experimental results closely align with theoretical expectations, confirming high-fidelity imaging without requiring a large sensor. Dual-mode functionality enables both rapid assessments and detailed, large-area imaging, enhancing its applicability in biological research.

**Conclusions:**

This compact and scalable imaging system overcomes the traditional magnification-FOV trade-off, offering a powerful tool for drug screening, cellular dynamics studies, and microfluidic-based biological analyses. Its high-resolution capability and adaptability make it a valuable asset for advancing OoaC technologies.

## 1 Introduction

Compact, high-resolution and high-throughput imaging systems capable of imaging large fields of view (FOV) at single-cell resolution are increasingly essential for biomedical research, particularly in applications involving live-cell assays, real-time monitoring of cellular dynamics, drug response assessments, and cell migration studies. The need for combining these features is highlighted by recent advances in multimodal imaging,^1^ lensless imaging,^2^ Fourier ptychographic microscopy,^3^ and wide-field fluorescence imaging;^4^ however, each method typically addresses only a subset of these requirements. Meanwhile, conventional microscopy methods face intrinsic limitations arising from the fundamental inverse relationship between magnification and field size. Capturing extensive areas with adequate magnification presents significant challenges for single-channel imaging systems, as higher magnifications inherently limit the observable field size. Specifically, increasing the FOV in a single-channel optical system typically pushes the design away from the paraxial regime, leading to significant optical aberrations and complexity in optical design.^5^ Alternatively, employing mechanical scanning to expand coverage introduces considerable mechanical complexity, prolongs imaging times, and potentially disrupts sensitive biological samples.

Multichannel systems employ, instead of single lenses, microlens arrays (MLAs), effectively partitioning the entire FOV into segments, each independently imaged by a distinct channel within the system. This design significantly reduces the FOV required for each optical sub-system, diminishing the impact of off-axis aberrations on the overall image quality. On the other hand, a drawback when using a multichannel system is the image overlap, also referred to as crosstalk, between adjacent channels.^6^ A common approach to mitigate the inter-channel crosstalk is to use MLA of low numerical aperture (NA) and thus low-resolution, combined with small working distances. A complementary strategy employs baffles to block rays that lie outside each sub-system’s intended field of view. However, this method is generally more effective in fluorescence imaging,^7^ where labeled regions are typically sparse, reducing the likelihood of significant crosstalk. Under these constraints, MLAs have been used for imaging in applications where the sensor is close to the object, such as projection setups,^8^ head up displays,^9^ integral^10^ and light field imaging,^11^ but rarely extended to high-magnification, large-FOV live-cell applications.

In this work, we introduce a novel multichannel imaging system that employs MLAs, micro-aperture arrays, and a micro-scanning approach to fully eliminate crosstalk while achieving both wide FOV and high magnification. By combining commercially available MLAs with laser-fabricated micro-aperture arrays, our prototype captures an effective FOV of $∼50\;{\mathrm{mm}}^{2}$ at 4× magnification, roughly an order of magnitude larger than a conventional 4× microscope objective yet maintains single-digit micron resolution. Notably, the system also features a working distance nearly 10× greater than typical MLA-based setups.^7^ Those capabilities are particularly advantageous in live-cell imaging contexts requiring both wide coverage and cellular detail (e.g., organ-on-a-chip studies), whereas the extended working distance is essential for imaging cells within microfluidic channels in such platforms. Moreover, we demonstrate that our system operates under two distinct modes:

Rapid sampling mode, which acquires immediate snapshots from each channel without stitching, ideal when only partial coverage across large fields is needed for rapid screening or monitoring specific regions over time.

Full-field imaging mode, which implements micro-scanning and image stitching to construct a continuous, detailed view across the entire synthetic field ($8.4\times 6\;{\mathrm{mm}}^{2}$ in our setup).

We validate the system by imaging live Lewis lung carcinoma (LLC) cells in an organ-on-a-chip platform, where the dual-mode flexibility proves valuable for both quick checks of cell distribution and comprehensive mapping of the entire chip for in-depth analysis. Beyond live-cell work, the same architecture has broad potential in drug screening, toxicology, and high-throughput industrial inspections, thanks to its compact footprint (<40  mm total track length) and straightforward scalability (adding more channels can further expand the FOV).

Altogether, this dual-mode multichannel design offers a robust solution to the magnification–FOV dilemma, providing a path to high-throughput yet high-resolution imaging for a diverse range of biological and engineering applications. The following sections detail our system’s optical design, the micro-scanning methodology, and the experimental tests confirming its performance and suitability for real-world live-cell imaging challenges.

## 2 Method

### 2.1 Multichannel Architecture and Micro-Scanning Principle

Multichannel imaging systems work by segmenting the FOV into smaller parts, with each one imaged by one of the system channels. The complete image is then retrieved by stitching all the sub-images together. In this work, we have developed a multichannel imaging system operating at 4× transverse magnification that is based on a cascade of 2 MLAs. To address the optical crosstalk between the adjacent channels, we use micro-aperture arrays that block rays from crossing from one channel to the other. Such an approach has been demonstrated^6^^,^^12^^,^^13^ in MLA relay system configurations, which compared with our system, were limited to low transverse magnification (1× or less), narrow FOV per channel (corresponding to the surface of the corresponding lenslet) and very limited working distances. When such a system is designed to achieve a magnification higher than 1×, the images generated by each channel will overlap on the image plane.

**Fig. 1:**
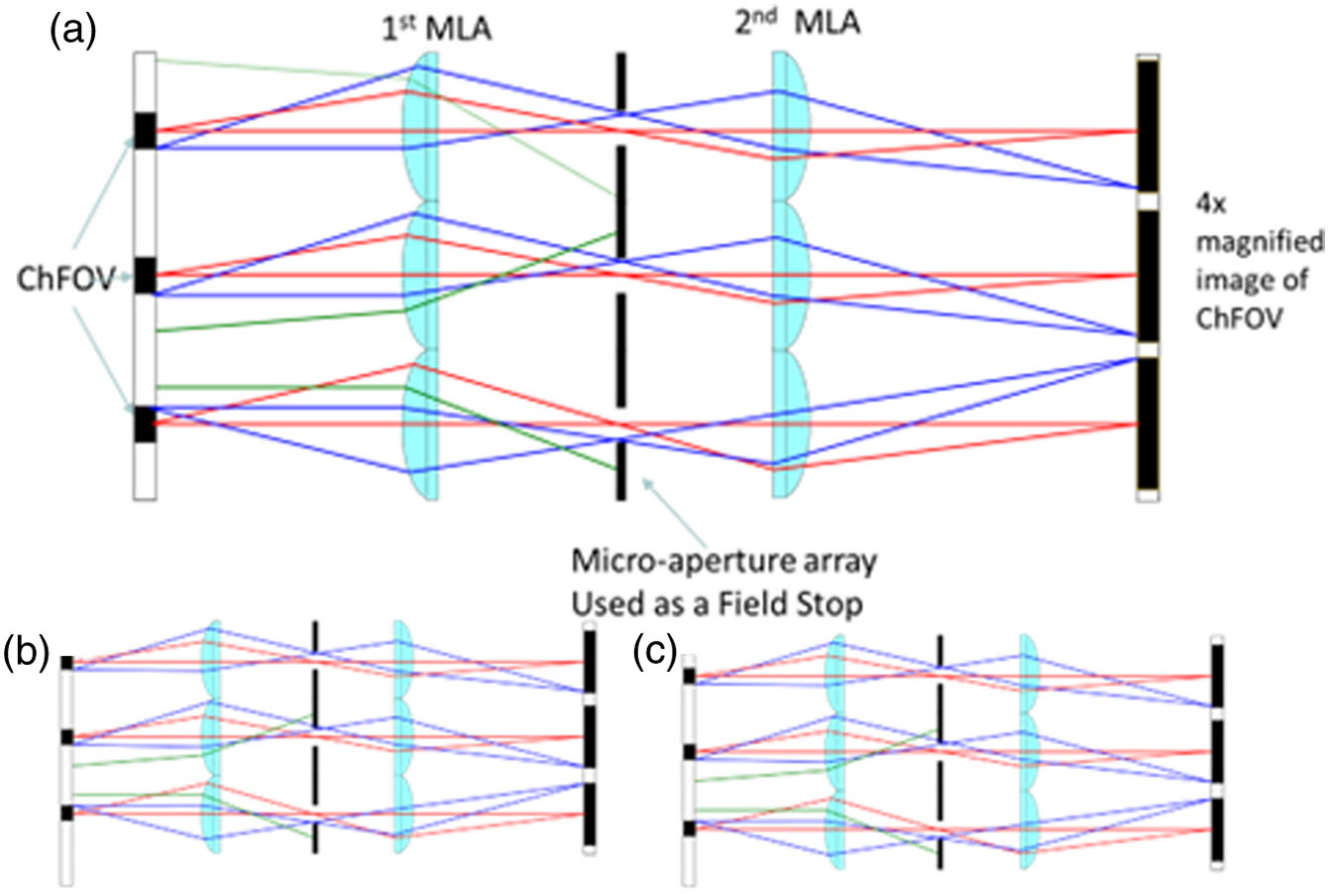
(a) Graphic depiction of a multichannel imaging system (3 channels depicted) with the use of a micro-aperture array as a field stop placed on the intermediate image of the system. Black regions on the object plane refer to the ${\mathrm{FOV}}_{\mathrm{ch}}$. The optical system operates at 4× magnification, and ${\mathrm{FOV}}_{\mathrm{ch}}=1/16$ of the lenslet area. (b), (c) visualization of 2 out of 5 scanning steps.

In this study, we employ a micro-aperture array that prevents crosstalk by obstructing light rays emanating from adjacent channels. Furthermore, the micro-aperture array is designed to restrict the view (FOV) of each channel in such a way that image overlap, even at higher magnification, is avoided. The basic operation of such a system is depicted Fig. 1(a), where a simpler, 3-channel system is visualized. The micro-apertures in the array limit the FOV of each channel (${\mathrm{FOV}}_{\mathrm{ch}}$), acting as a field stop. Only the black shaded regions in the object plane will be imaged, whereas rays originating from other regions are blocked. The drawback in this approach is that blind spots are formed between the channels (denoted as white regions in the object plane). To overcome this, we use micro-scanning, as shown in Fig. 1(b).

In a conventional scanning approach (a microscopy setup mounted on a translational stage), the microscope translates across the sample in steps equal to its total FOV. By contrast, micro-scanning confines the stage motion to the area covered by a single channel, making the process significantly faster and more efficient. Consequently, the system’s total FOV is simply the product of the number of channels and the area of each channel. Likewise, as the micro-aperture array is placed on a plane where an intermediate image is formed, it functions as a field stop that determines the ${\mathrm{FOV}}_{\mathrm{ch}}$ in the following way:

$${\mathrm{FOV}}_{\mathrm{ch}}\leq {\mathrm{FOV}}_{\max}=\frac{A}{M_{T}^{2}},$$ (1)

where ${\mathrm{FOV}}_{\max}\equiv \max\{{\mathrm{FOV}}_{\mathrm{ch}}\}$, A is the channel area and $M_{T}$ is the transverse magnification. Also, in our imaging system each channel can image an area of the object that is centered along the optical axis of each lenslet, whereas the area A corresponds to the lenslet’s area. In such a way in a system of transverse magnification $M_{T}=4$ and square lenslets with 1 mm sides, the FOV of each channel is ${\mathrm{FOV}}_{\mathrm{ch}}\leq 0.0625\;{\mathrm{mm}}^{2}$. To access the blind spots, the object is translated in reference to the optical system Figs. 1(b) and 1(c). The total steps needed are defined as

$$\text{Steps}=\frac{A}{{\mathrm{FOV}}_{\mathrm{ch}}}=M_{T}^{2}.$$ (2)

Using this approach, optical systems can be adapted to operate at a desired traverse magnification $M_{T}$ value without crosstalk. On the other hand, as $M_{T}$ is increased, more steps are needed to cover the whole FOV, whereas the total movement of the translation stage remains the same.

### 2.2 Dual-Mode Imaging

Our system is designed for two distinctive operational modes; rapid sampling mode for instantaneous, partial coverage imaging, and full-field imaging mode for comprehensive stitched imaging.

#### 2.2.1 Rapid sampling mode

This mode captures instant snapshots of partial regions across the sample’s overall field. No stitching is performed, so each channel directly images its sub-region leaving gaps between channels. This is useful when only random or targeted regions of the sample need quick inspection (e.g., early screening steps, detecting broad changes, or monitoring multiple zones at once). By bypassing micro-scanning, it offers high-speed data acquisition across a wide area, albeit without covering every point in the sample.

#### 2.2.2 Full-field imaging mode

When the application requires detailed visualization of the *entire* sample, we employ the micro-scanning procedure outlined in Sec. 2.1. The sample is translated in discrete steps so that the system images all blind spots between adjacent channels. After acquisition, the sub-images are stitched together computationally to produce a continuous, large-area image at 4× magnification. Although this approach takes more time due to the scanning and stitching steps, it ensures that every region within the $8.4\times 6\;{\mathrm{mm}}^{2}$ (synthetic) field is captured at the system’s native resolution. By switching between these two modes, users can balance speed versus completeness. Rapid sampling mode is ideal for quick overviews of large samples, whereas full-field imaging mode addresses the need for exhaustive coverage and high spatial resolution across the entire sample area.

## 3 Optical Design

The optical design process for our multichannel imaging system was organized into two phases. In the initial phase, we studied and optimized a single channel which served as a prototype for the other identical channels in the system. During this phase the channel optics were designed, its performance was analyzed and optimized using traditional optical design methodologies.^14^ In the second phase, the complete multichannel system was simulated allowing us to assess the channels interaction and evaluate the final image quality. This two-step design process allowed us to isolate and study independently crucial aspects of the multichannel imaging system.

### 3.1 Design of a Single Channel

As the system consists of an array of identical channels, our analysis first focuses on optimizing a single channel with respect to optical performance. The target magnification for the multichannel imaging system was $M_{T}=4$, so each single channel should be designed to offer such a magnification. The optical configuration of the single channel comprises 2 lenses. The first creates an inverted image of the object with a $m_{1}=0.8$ magnification and the second an $m_{2}=5$ magnification, as a result the total magnification, M, of the system is

$$M_{T}=m_{1}\cdot m_{2}.$$ (3)

**Fig. 2:**
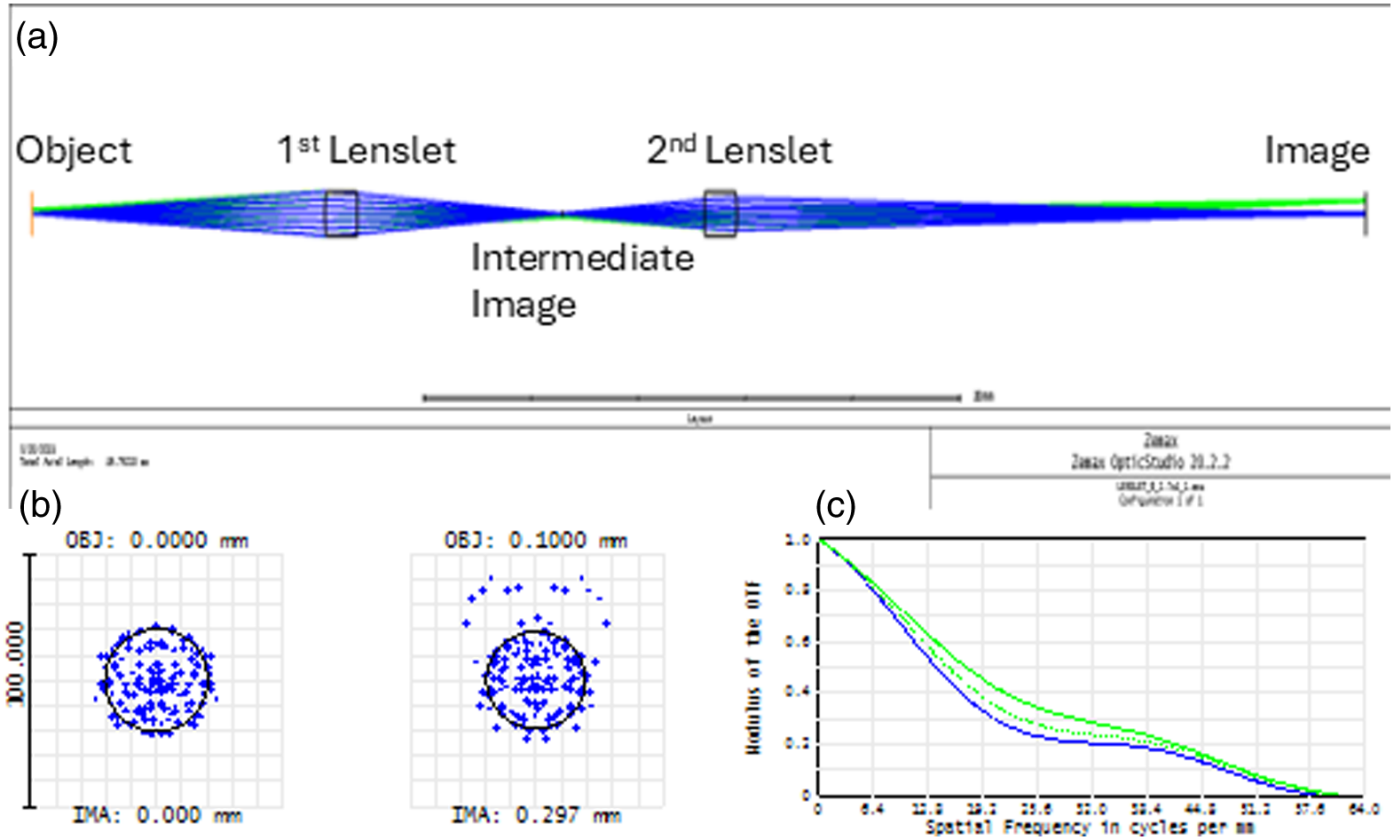
(a) 2D Layout and raytracing of a single channel of the designed system (b) Spot diagram and (c) MTF. The results are presented for 2 object points (on axis, $y_{0}=0\;\mathrm{mm}$, and at the edge of channel’s field of view at $y_{0}=0.1\;\mathrm{mm}$).

The designed system is comprised of 2 identical lenslets with effective focal length EFL= 4.7 mm and diameter D=1  mm. The above specifications were chosen so that the system could be fabricated using commercially available MLAs. In Fig. 2(a), the 2D Layout of one channel is presented. The maximum field of view in this case is estimated by Eq. (1) to $OV_{\max}=0.0625\;{\mathrm{mm}}^{2}$. To avoid channel crosstalk from image overlapping and to obtain a clearer image a value of ${\mathrm{FOV}}_{\mathrm{ch}}=0.04\;{\mathrm{mm}}^{2}$ was chosen. The aperture diameter to achieve this ${\mathrm{FOV}}_{\mathrm{ch}}$ is $D_{a}=0.16\;\mathrm{mm}$.

The spot diagram for two object points, located on axis and at the edge to of the channel’s field of view, is presented in Fig. 2(b) The RMS radii for the 2 fields are 16.1 and 18.5  μm, respectively. Under this design scheme, the object space NA for the single-channel system is ${\mathrm{NA}}_{\mathrm{ch}}\approx 0.05$ and the corresponding Airy disc is 20.3  μm, so the spot diagrams suggest that this system is close to diffraction limited. Although the spot diagrams accurately depict the points where rays intersect with the focal plane, no information is provided about the focal intensity distribution and thus the actual spot size. As shown in Fig. 2(c), the modulus of the optical transfer function (OTF) quantifies in more detail the single-channel performance. Taking as a threshold the OTF value of 0.2 (corresponding to 20% contrast), we get that the resolution on the image plane is ∼38.4  lines/mm. Therefore, the resolution on the object plane, considering that the system works at 4× magnification is estimated to be 153.6 lines/mm, corresponding to a spot size of ∼6.5  μm.

### 3.2 Crosstalk and Design of a Larger Sub-system

**Fig. 3:**
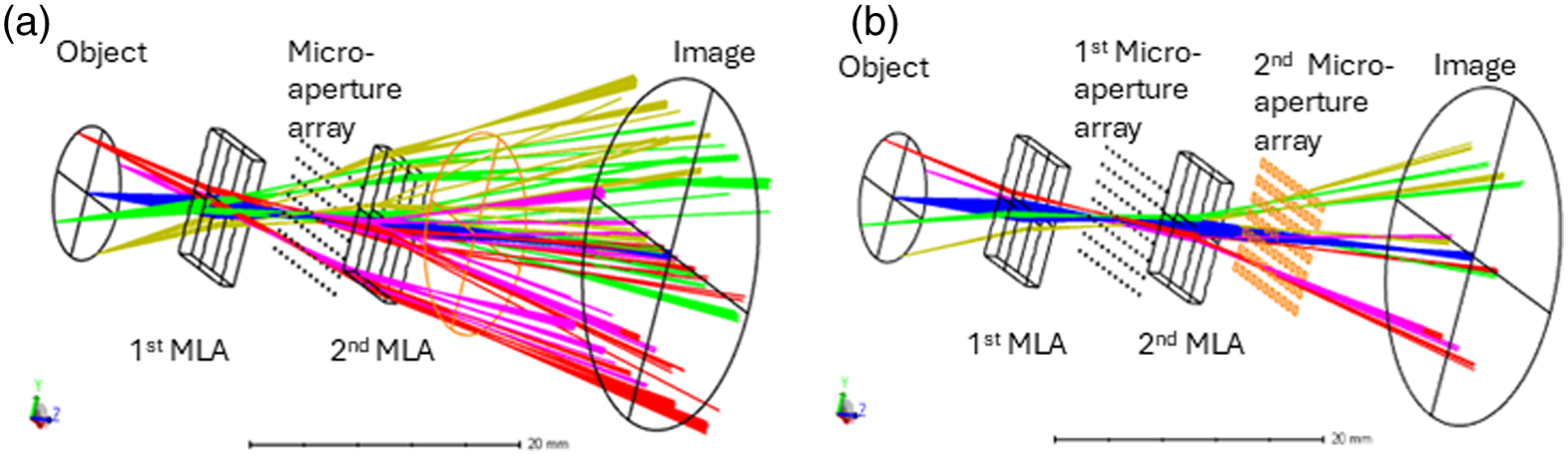
3D Layout of the multichannel system. (Note that the micro-aperture array openings are denoted as black rectangles). (a) Single micro aperture array at intermediate image, (b) Two micro aperture arrays at intermediate image and exit pupil.

To study the effect of crosstalk between adjacent channels, we simulated and analyzed a system comprised by an array of 6×6 single channels. In our design, as shown in Fig. 3, the aperture of each lenslet is rectangular, of dimensions 1×1.4  mm.

**Fig. 4:**
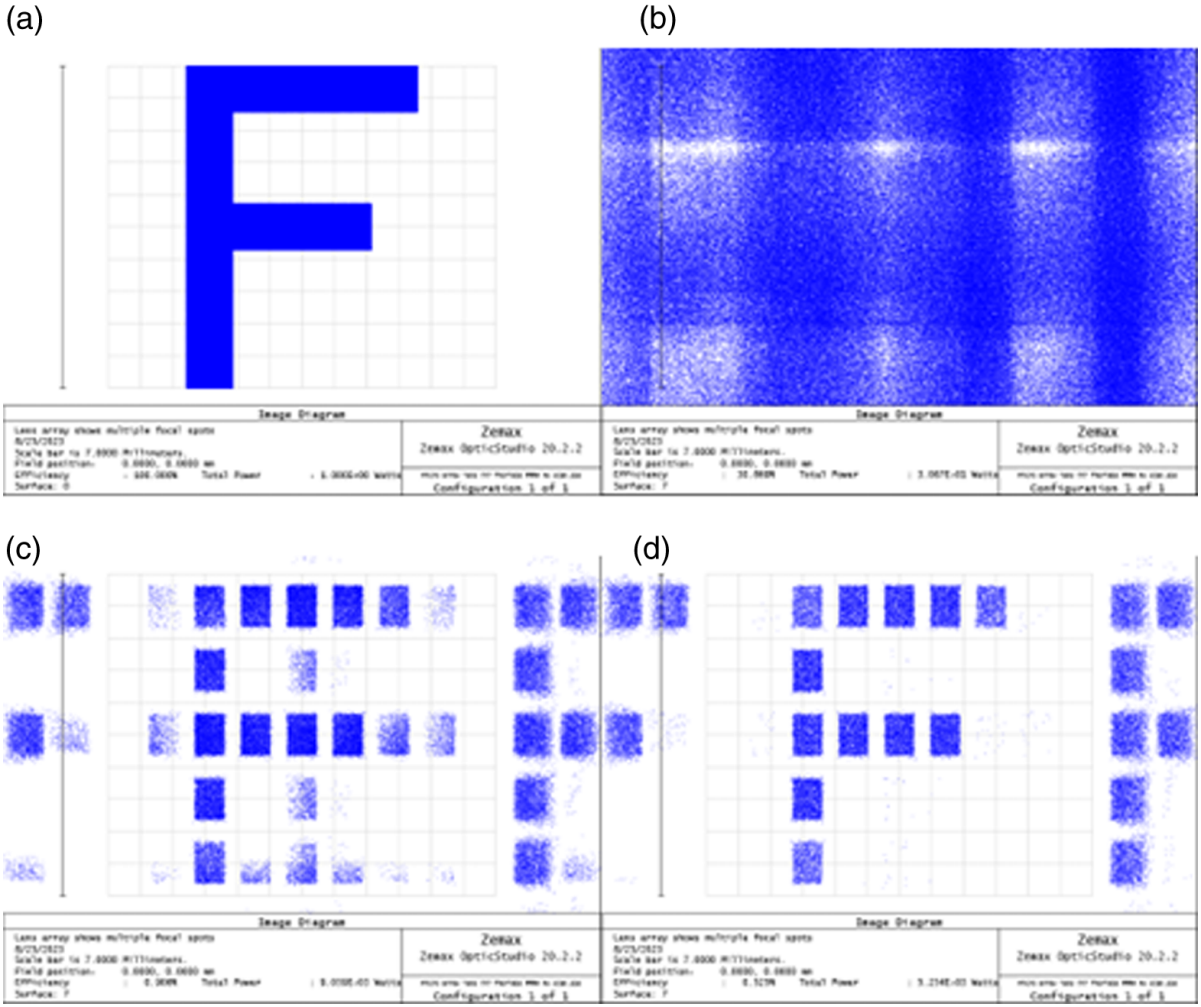
Imaging performance of the array. Simulations were performed using Zemax imaging analysis. (a) Object used for image simulations, (b) system with no micro-apertures, (c) system with 1 micro-aperture, and (d) system with 2 micro-apertures.

We tested the imaging performance of the array using the letter F, shown in Fig. 4(a), as an object.

The first simulation used a system with 2 MLAs but no micro-aperture array Fig. 4(b). Clearly, channel crosstalk leads to sub-image overlapping that hinders the identification of any structure in the final image. In the second simulation shown in Fig. 4(c), and described in detail in the method section, an array of identical micro-apertures is introduced as a field stop. Each micro-aperture in this array has dimensions of 160×224  μm. The micro-aperture array was placed on the plane of the intermediate image filtering out some crosstalk so now the letter F image can be identified in the final image. Some residual ghost rays can be seen in the image; however, their intensity is low. Note that the image is segmented as, to avoid crosstalk, we have chosen a transverse magnification of $M_{T}=4$ that leads to a ${\mathrm{FOV}}_{\mathrm{ch}}=0.04{\;\mathrm{mm}}^{2}<{\mathrm{FOV}}_{\max}$. By placing a second micro-aperture array on the plane of the exit pupil, the residual ghost rays are eliminated, and image is further cleared as shown in Fig. 4(d). Finally, to access the blind areas of the image, and retrieve a complete image, the micro-scanning procedure, as described in detail in Sec. 2, is implemented.

## 4 System Implementation

**Fig. 5:**
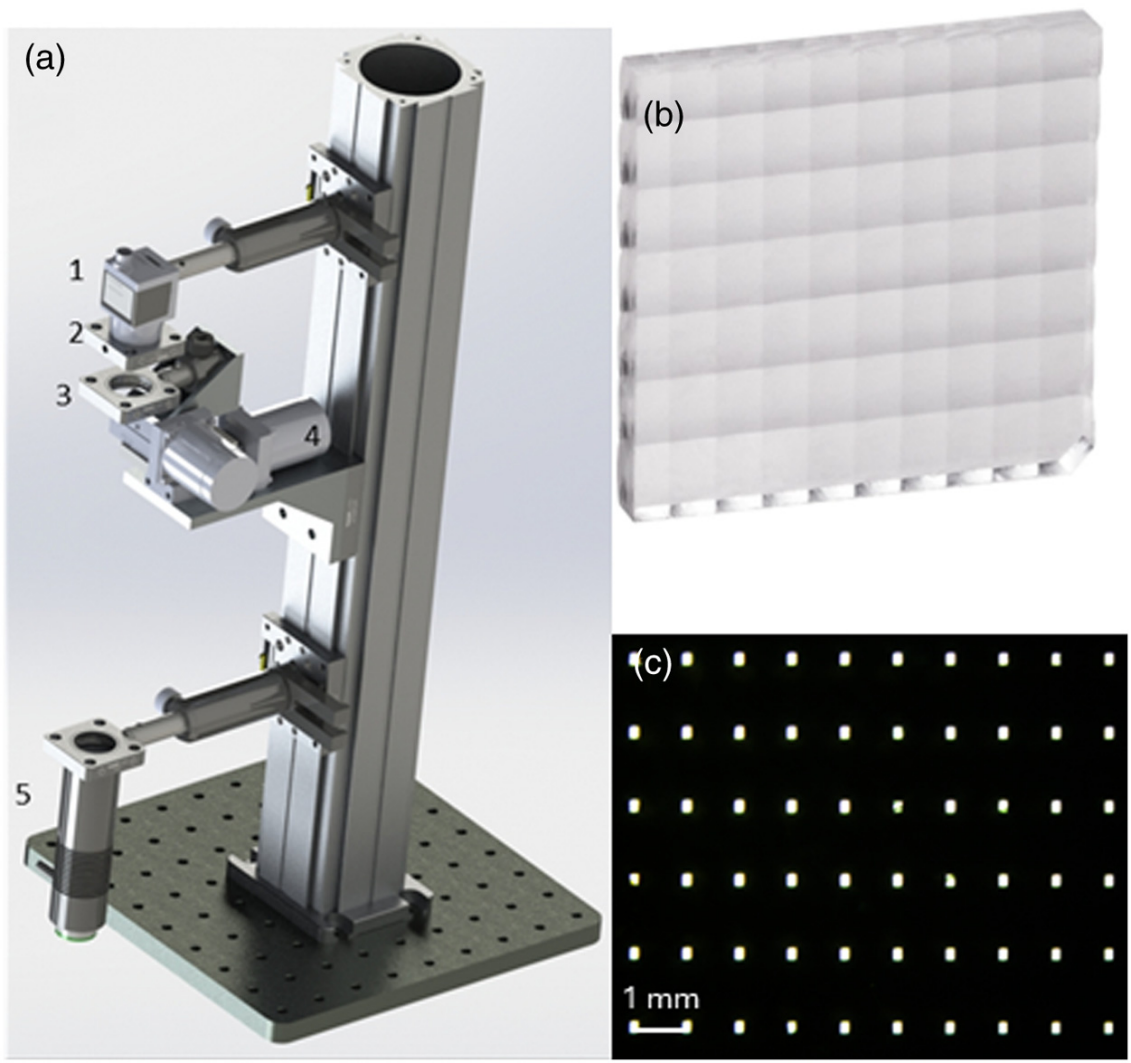
(a) System design: (1) sensor, (2) imaging system, (3) sample holder, (4) translational stage, and (5) illumination system. (b) MLA. (c) Microscope image of the micro-aperture array (trans-illuminated).

The optomechanical design of the system is presented in Fig. 5(a). The micro-scanning is achieved by mounting the sample holder on an automated xy translational stage (THORLABS M30XY/M). In this way, the sample is moved relative to the imaging and illumination systems, which are fixed in position. The imaging module was constructed using two identical, commercially available, MLAs (THORLABS MLA1M1, 7×10 lenslets), shown in Fig. 5(b), and a micro-aperture array, shown in Fig. 5(c). The micro-aperture array was fabricated in-house by using laser ablation on a silver-coated glass substrate. The illumination system comprised a green LED (Thorlabs M530L4) paired with an aspheric condenser (Thorlabs ACL2520U-DG6-A) to collimate the light. Overall, the imaging module consisted of 36 channels (6×6 channels) selected from the 7×10 available, simplifying the alignment procedure.

## 5 Experimental Results

### 5.1 Validation of the System

**Fig. 6:**
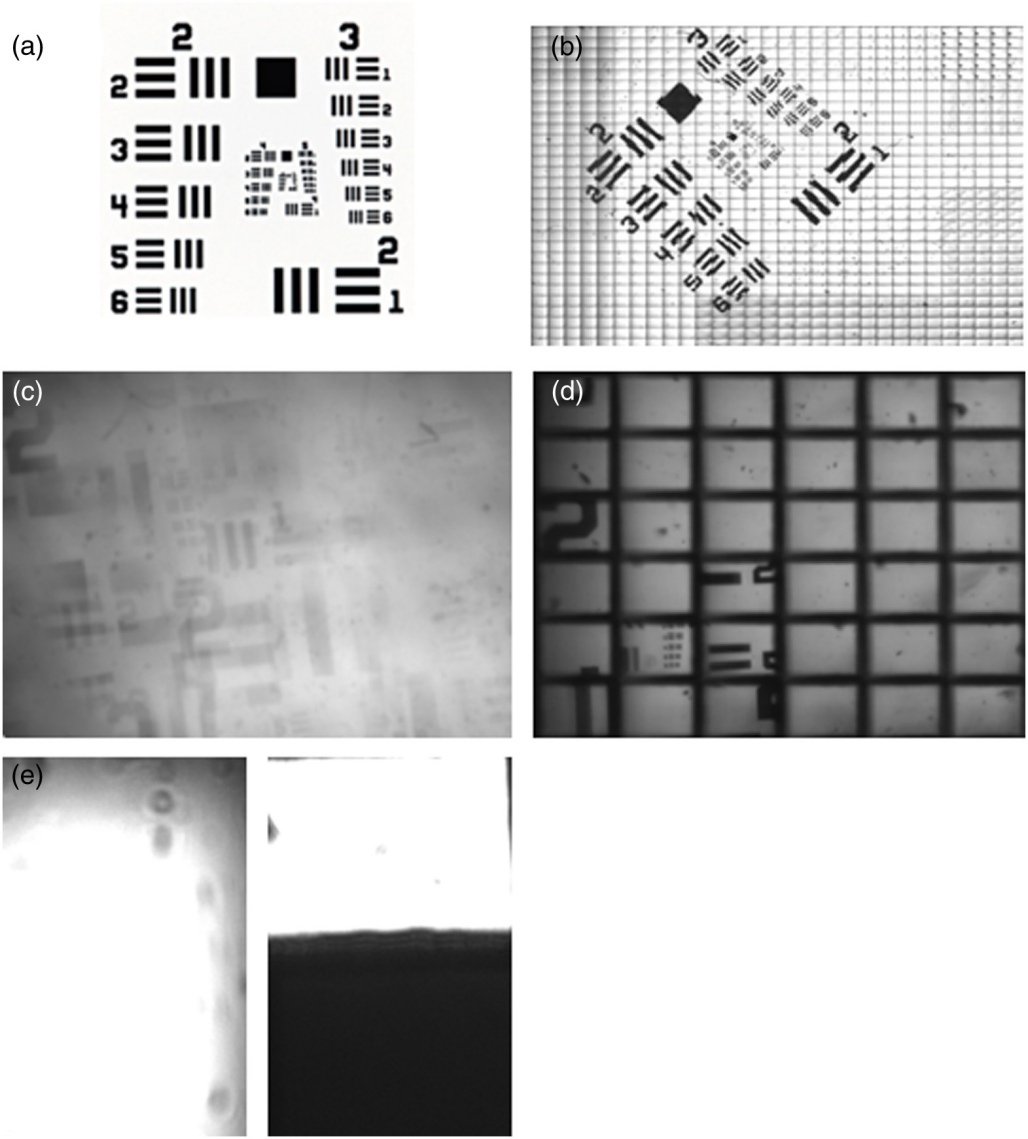
(a) USAF 1951 resolution target, (b) full-field image of the USAF 1951 resolution target, (c) image produced by the developed system with no aperture arrays, (d) image produced by the developed system with 1 aperture array, and (e) image of a pinhole 2 adjacent channels, (left) no-apertures (right) 1 aperture array.

Employing the system we engineered, we proceeded to validate it with respect to the specifications described in Sec. 3. First, to check the optical quality of the imaging module, we used a resolution target, shown in Fig. 6(a) as an object. In Fig. 6(c), the image was retrieved without incorporating the micro-aperture array in the imaging module. As expected, noticeable image overlapping from channel crosstalk is observed. Incorporating the micro-aperture in the imaging module, a significant enhancement in image quality is observed, as shown in Fig. 6(d). The incorporation of the micro-aperture array effectively mitigates the channel overlapping and yields distinct and clear images for each channel (depicted as rectangular illuminated areas), achieving full optical isolation. The FOV for each channel is $280\times 200\;\mu m^{2}$. Both experimental results agree with our raytracing simulations, verifying that the micro-aperture array can effectively reduce the channel crosstalk. Note that Fig. 6(d) depicts the images captured by our system when working in the rapid sampling mode, to image the complete FOV the micro-scanning technique is used Fig. 6(b).

To quantify inter–channel crosstalk, we imaged a bright–field pinhole sized to fill a *single* microlens channel at the specimen plane. For each acquisition we defined a primary ROI (illuminated channel) and neighbor ROIs in the two immediately adjacent channels. Mean pixel intensities (gray level) were background–subtracted (dark ROI) to yield $I_{\mathrm{prim}}$ and $I_{\mathrm{neigh}}$. Crosstalk was calculated as

$$\mathrm{CT}=\frac{I_{\mathrm{neigh}}}{I_{\mathrm{prim}}}$$ (4)

Without the micro–aperture array we measured $I_{\mathrm{prim}}=202$ and $I_{\mathrm{neigh}}=195$ giving ${\mathrm{CT}}_{\text{no-}\mathrm{ap}}=1.035$. With the aperture array in place, we obtained $I_{\text{prim}}=238$ and $I_{\mathrm{neigh}}=12$ giving ${\mathrm{CT}}_{\mathrm{ap}}=19.83$. Thus, the apertures reduce inter–channel leakage by ∼19- to 20-fold.

### 5.2 Image Quality

**Fig. 7:**
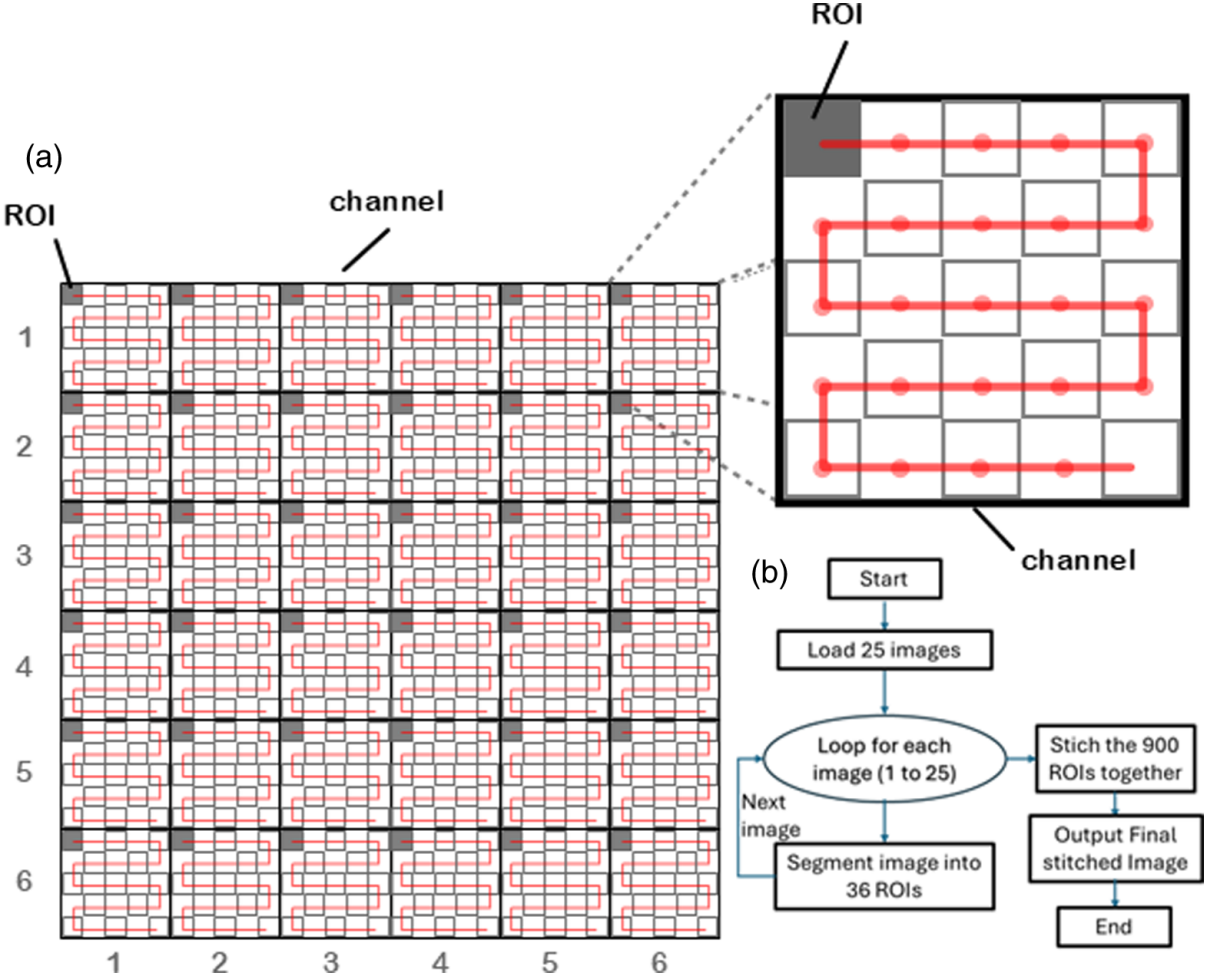
(a) Visualization of the micro-scanning process geometry for the entire FOV. (b) Visualization of the micro-scanning steps within the area of a single channel. (c) Flow chart of the numerical algorithm.

To demonstrate the complete FOV of the imaging module, we incorporated the micro-scanning technique. The process was completed by the following steps:

• The FOV of each channel is divided into a 6×6 array of sub-images. By moving the sample, a total of 25 images were captured, covering simultaneously all channels as shown in Fig. 7(a). The visualization of the scanning along 1 channel is shown in the inset of Fig. 7(a).
• From each image, 36 regions of interest (ROIs) were identified by isolating, using a numerical algorithm implemented in Python, the illuminated areas that correspond to the FOV of each channel.
• By combining the ROIs of all images, a total of 36×25=900 ROIs were identified and then stitched together, using a numerical algorithm implemented in Python, Fig. 7(b), to form the final image.

**Fig. 8:**
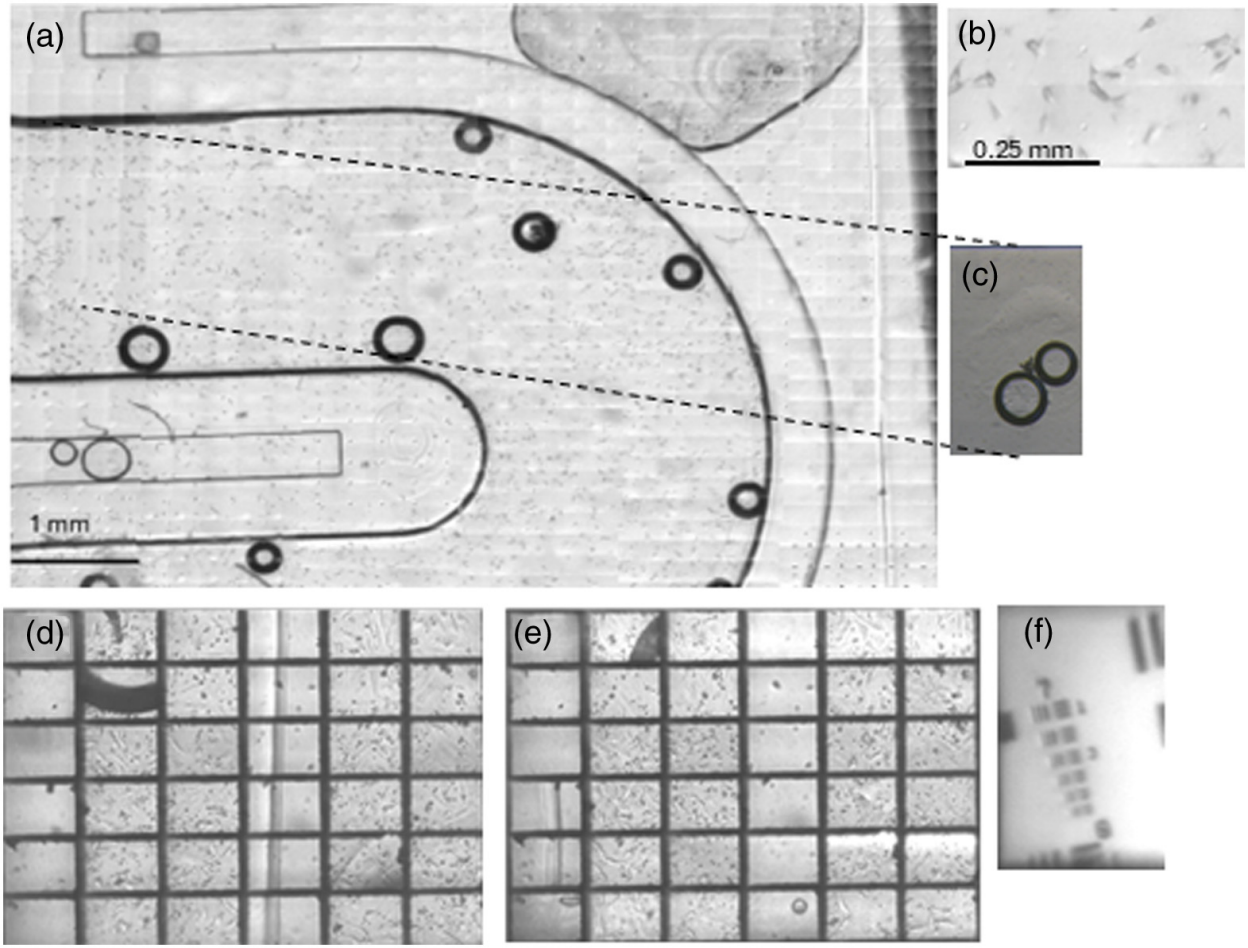
(a) Image of an organ-on-a-chip platform demonstrating the large FOV of the full-field imaging mode. (b) Magnified area of image (a) where the LLC cells are clearly visible. (c) Image from a conventional brightfield microscope using a 4x objective. (d), (e) Images from rapid sampling mode. (f) Image of a USAF 1951 resolution target.

Using the developed system and the method described in the current section the USAF 1951 resolution target Fig. 6(a) was imaged. By inspecting the zoomed in part of an image featuring a USAF 1951 resolution target Fig. 8(f), where the 3rd element of the 7th group was imaged with barely distinguishable lines, we estimated the system’s cutoff frequency to be 161  lines/mm.

Consequently, the imaging module system achieves a resolution of 6.2  μm, and lines of 3.1  μm can be resolved, in agreement with the ray tracing simulation results.

Likewise, using the described process, we imaged an organ-on-a-chip platform filled with LLC (Lewis lung carcinoma) cells in both working modes of the system. LLC cells (sourced from the Biomedical Research Foundation of the Academy of Athens, Greece) were chosen due to their consistent growth behavior and typical size range in suspension (15 to 20  μm). The cells were maintained under standard culture conditions in high-glucose Dulbecco’s Modified Eagle Medium (DMEM; SH30243.01, Cytiva, USA), supplemented with 10% fetal bovine serum (FBS; FBS-12A, Capricorn Scientific, Germany) and 1% penicillin/streptomycin (15140–122, Gibco, Thermo Fisher Scientific, USA). Cultures were incubated at 37°C in a humidified atmosphere containing 5% ${\mathrm{CO}}_{2}$.

Once the cultures reached roughly 90% confluency, the monolayers were washed with phosphate-buffered saline (PBS) to eliminate any remaining medium and then detached by treatment with trypsin (T4049, Sigma-Aldrich, USA) for 1 to 2 min at 37°C. The enzymatic activity was stopped by adding DMEM containing 10% FBS, followed by centrifugation at 250 g for 5 min to collect the cells.

The resulting cell pellet was resuspended in fresh medium to achieve a final concentration of 1 million cells per milliliter. Cell density was measured using a Neubauer hemocytometer, with volume adjustments made accordingly for accuracy.

The prepared cells were then introduced into a microfluidic chip developed within the framework of the European project Tumor-LN-oC and incubated at 37°C for 24 h before imaging. In Figs. 8(d) and 8(e), two representative images from rapid sampling mode are shown, where each rectangular region of interest (ROI) has a field of view (FOV) of $280\times 200\;\mu m^{2}$, spanning an overall $8.4\times 6\;mm^{2}$ portion of the sample, the distance between the centers of 2 adjacent ROIs (in object space) is 1 mm in the x direction and 1.4 mm in the y.

The full-field imaging mode result is depicted in Fig. 8(a), displaying a continuous, stitched FOV of $8.4\times 6\;mm^{2}$ with high resolution (≈145  Mega Pixels). A large number of LLC cells are clearly visible and well resolved, as illustrated in Fig. 8(b), which shows a magnified portion of the stitched image. The approximate time for acquiring a Full-Field image is 1 minute. The working distance of our implemented system is 14 mm. Both modes were employed together to monitor such events: rapid sampling mode was used continuously to provide constant observation of cells, whereas full-field mode was used at predetermined intervals to obtain a comprehensive view of the system under study. Figure 8(c) shows a reference image of the LLC-cell–seeded organ- on-a-chip device acquired with a conventional wide-field microscope and a 4× objective, comparable single–cell detail is visible, but only within a much smaller field of view $2.2\times 1.5\;{\mathrm{mm}}^{2}$ on a 2/3 format sensor. We quantitatively compared image quality using background signal-to-noise ratio (SNR) and pooled contrast-to-noise ratio (CNR),^15^ two metrics that capture noise cleanliness and feature detectability. SNR was computed on structure-free background patches as $SNR_{bg}=\mu_{bg}/\sigma_{bg}$ indicating how clean the background is. Pooled CNR was computed on paired cell and local-background ROIs as

$$\mathrm{CNR}=\frac{\left|\mu_{\text{cell}-\mu_{bg}}\right|}{\sqrt{\sigma_{\text{cell}}^{2}+\sigma_{bg}^{2}}}$$

which reflects the visibility of a cell relative to the combined noise of cell and background. For a fair comparison, both datasets were matched in object-space sampling (same μm/px) and used 20×20  μm ROIs. We analyzed n=7 cell/background pairs per system sampled across the field and report mean ± SD. The results are presented in Table 1. As expected, the 4× microscope exhibits a cleaner background (higher SNR), whereas our multichannel Full-Field mode provides higher pooled CNR, i.e., stronger per-cell detectability at matched scale.

**Table 1:** Quantitative comparison of a Wide-field microscope and the Multichannel System.

Metric (mean ± SD; n = 7)	Wide-field microscope	Dual-Mode MLA
${\mathrm{SNR}}_{bg}$	125.68 ± 16.72	83.61 ± 21.85
CNR	0.45 ± 0.07	1.78 ± 0.45

These results show that although the conventional 4× objective delivers a very clean background, the proposed multichannel architecture preserves and, in these tests, improves cell-level detectability, supporting its suitability for large-FOV monitoring.

Because the stitched field in Fig. 8 spans 8.4×6  mm, a conventional stage-scanning microscope equipped with a 4× objective must translate the specimen in increments equal to its native frame size (2.2 mm in x and 1.5 mm in y) producing a 4×4 mosaic of 16 tiles, if no overlapping is used. Our micro-scanning approach, by contrast, advances the imaging array by just 0.28 mm (x) and 0.20 mm (y) per step. The difference in total mechanical travel underscores the speed advantage of our approach. To cover the full field, a conventional stage scan must translate 30.9 mm, whereas the micro-scanning mechanism moves only 6.4 mm. This roughly fivefold reduction in displacement leads to much shorter move and settle times per tile, so the full field can be imaged markedly faster while still preserving single-cell resolution across the entire area. Achieving high resolution of individual cells across a wide FOV is essential in organ-on-a-chip platforms because it enables the simultaneous visualization of large sections, or even the entirety, of the platform to capture all migration events.

## 6 Discussion

**Fig. 9:**
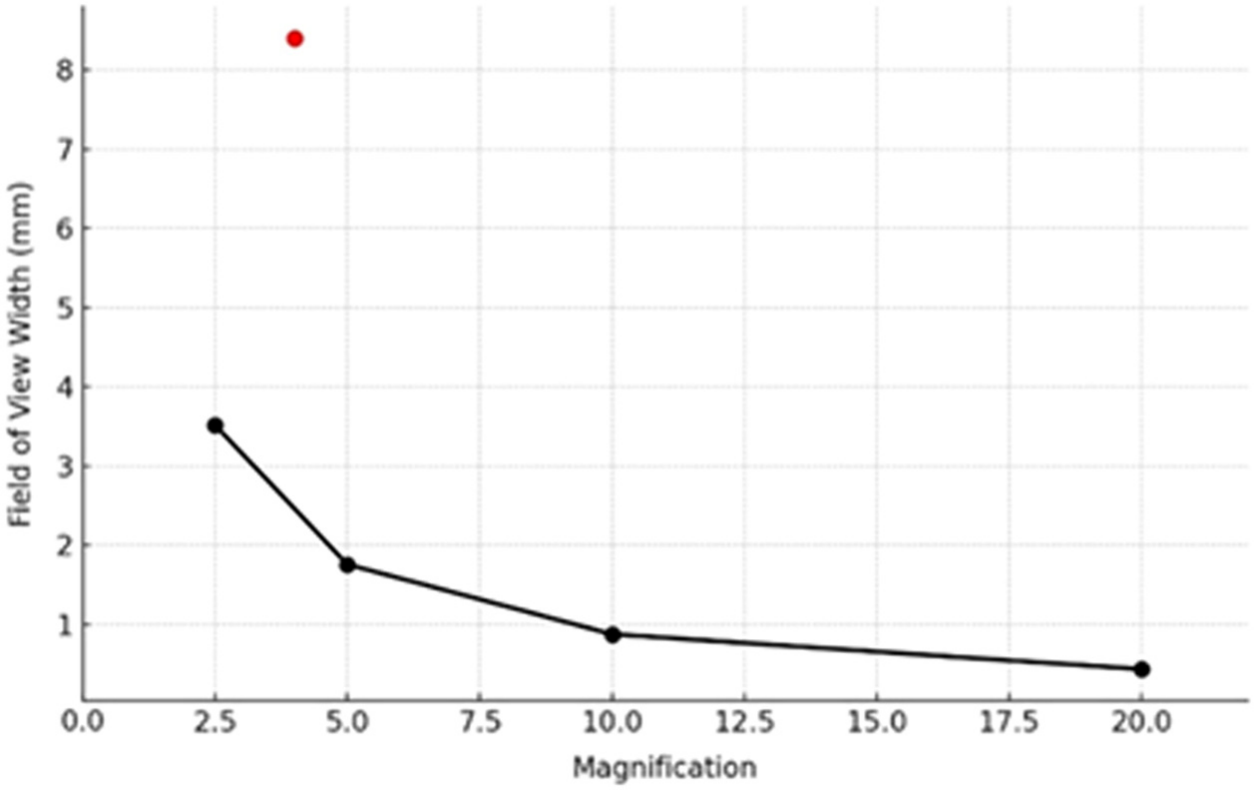
FOV versus Magnification for conventional microscopy objectives^16^ on 2/3″ format sensor, the red dot indicates the multichannel system performance.

We have successfully demonstrated that our 36-channel imaging system achieves a synthetic field of view (FOV) of $8.4\times 6\;mm^{2}$ at a 4× magnification and a resolution of 6.2  μm. This performance notably transcends the traditional limitations observed in conventional microscopy, where FOV and magnification are inversely proportional. This is illustrated in Fig. 9, where the linear FOV of various imaging systems is depicted as a function of the system magnification, as well as in the quantitative analysis in Table 1. Compared with typical microscope objectives, our multichannel synthetic FOV approach shows that the FOV can be decoupled from magnification. Another key aspect of our multichannel system design is its inherent scalability, as the FOV is proportional to the aggregate area covered by the channels. Thus, from the optical design perspective, we can directly expand the FOV by increasing the number of channels.

In addition to covering a large FOV at high resolution, the system supports two distinct modes: (1) a rapid sampling mode for fast, partial coverage imaging and (2) a full-field mode that utilizes micro-scanning to generate a continuous, stitched image. The flexibility provided by these dual modes allows users to balance speed against completeness depending on the application needs. For instance, in live-cell assays, rapid sampling can offer near real-time observation of select regions, whereas full-field imaging captures a complete layout of an organ-on-a-chip at single-cell resolution.

Another crucial aspect of our design approach is the compactness of the system. In conventional microscopy configurations, the imaging system typically includes an objective lens coupled with a tube lens. The total track length of the configuration varies within the range of 300 to 400 mm.^17^ By contrast, in our multichannel system design we significantly reduced, by a factor of ∼10×, the overall length to <40  mm (measuring from the first surface of the first element to the image plane). Compactness is a critical feature for a live-cell imaging system, as it must be adaptable to confined spaces, such as an incubator, without compromising functionality.

Recent MLA studies have tackled the FOV–versus–resolution trade-off with multichannel architectures similar to ours. Wang et al.^18^ achieved this by inserting an MLA, a set of objective/tube lens relays, and matching the NAs of the optical sub-systems, which suppressed inter–channel crosstalk. However, the resulting optical setup is bulky and not a pure MLA only solution so still is limited by the FOV of the main objective after the sample, $288\times 206\;{\mu m}^{2}$. Another approach^7^ incorporated the MLAs in a 4f setup. This approach worked only for fluorescence samples, where features are sparser, unlike bright-field images that are densely populated. In addition, the 4f setup significantly restricted the working distance, as previously explained. An alternative is the multicamera array microscope,^19^ which tiles 54 low-cost sensors with dedicated optics and relies on stage scanning to stitch gigapixel frames. Although this architecture achieves centimeter-scale coverage, it requires complex multicamera calibration, produces gigabyte-scale raw data, and its large footprint prevents integration in constrained environments. By contrast, our dual-mode MLA system provides comparable cell-level resolution over an 8.4×6  mm field using a single camera and 25 micro-scan steps while maintaining a 14 mm working distance compatible with organ-on-a-chip platforms. As a result, hardware complexity, size, data bandwidth, and cost are reduced by nearly two orders of magnitude.

Nonetheless, several limitations remain and define our immediate development roadmap. First, in full-field mode the acquisition rate is constrained by the mechanical micro-scans. Replacing the fixed micro-aperture array with a programmable element, such as an LCD mask that sequentially selects sub-regions, could deliver the full wide field without moving parts. Second, the current micro-aperture assembly is susceptible to external vibration. We therefore plan to introduce a stiffer, modular frame that offers additional adjustment degrees of freedom for the MLA, aperture plane, and sensor, thereby improving mechanical robustness and simplifying alignment.

## 7 Conclusion

In this work, we have successfully designed and developed a compact, dual-mode multichannel imaging system that overcomes the conventional trade-off between magnification and field of view (FOV). By combining MLAs, micro-aperture arrays, and a micro-scanning approach, our 36-channel prototype achieves a continuous FOV of $8.4\times 6\;mm^{2}$ at 4× magnification with a 6.2  μm resolution, far surpassing the constraints typical of single-channel optics. The system operates in two distinct modes: a rapid sampling mode for quick, partial coverage imaging and a full-field mode that stitches sub-images into one contiguous view. This design effectively decouples magnification from off-axis aberrations while preserving high image quality over large sample areas, and its inherent scalability allows the FOV to be expanded by adding more channels. Moreover, the overall track length is reduced by a factor of around 10× compared with conventional microscopy, facilitating straightforward integration into incubators, portable diagnostic devices, and other space-limited environments. Consequently, the dual-mode multichannel system holds strong promise for biomedical and industrial applications requiring high-resolution, wide-area imaging, such as live-cell assays, drug discovery, and cellular dynamics studies.

## Data Availability

The data that support the findings of this study are available from the corresponding author upon request.
